# Dose Dependent Effects on Cell Cycle Checkpoints and DNA Repair by Bendamustine

**DOI:** 10.1371/journal.pone.0040342

**Published:** 2012-06-29

**Authors:** Neil Beeharry, Jerome B. Rattner, Alfonso Bellacosa, Mitchell R. Smith, Timothy J. Yen

**Affiliations:** 1 Basic Science Division, Fox Chase Cancer Center, Philadelphia, Pennsylvania, United States of America; 2 Department of Cell Biology and Anatomy, University of Calgary, Calgary, Alberta, Canada; University of Chicago, United States of America

## Abstract

Bendamustine (BDM) is an active chemotherapeutic agent approved in the U. S. for treating chronic lymphocytic leukemia and non-Hodgkin lymphoma. Its chemical structure suggests it may have alkylator and anti-metabolite activities; however the precise mechanism of action is not well understood. Here we report the concentration-dependent effects of BDM on cell cycle, DNA damage, checkpoint response and cell death in HeLa cells. Low concentrations of BDM transiently arrested cells in G2, while a 4-fold higher concentration arrested cells in S phase. DNA damage at 50, but not 200 µM, was efficiently repaired after 48 h treatment, suggesting a difference in DNA repair efficiency at the two concentrations. Indeed, perturbing base-excision repair sensitized cells to lower concentrations of BDM. Timelapse studies of the checkpoint response to BDM showed that inhibiting Chk1 caused both the S- and G2-arrested cells to prematurely enter mitosis. However, whereas the cells arrested in G2 (low dose BDM) entered mitosis, segregated their chromosomes and divided normally, the S-phase arrested cells (high dose BDM) exhibited a highly aberrant mitosis, whereby EM images showed highly fragmented chromosomes. The vast majority of these cells died without ever exiting mitosis. Inhibiting the Chk1-dependent DNA damage checkpoint accelerated the time of killing by BDM. Our studies suggest that BDM may affect different biological processes depending on drug concentration. Sensitizing cells to killing by BDM can be achieved by inhibiting base-excision repair or disrupting the DNA damage checkpoint pathway.

## Introduction

Bendamustine (BDM) represents one of the earliest rationally designed anticancer drugs that incorporated three functional groups; a benzimidazole ring, a mechlorethamine group and a butanoic acid residue. These groups putatively endowed BDM with both alkylator and anti-metabolite activities. BDM has been found to be especially effective in hematologic-related cancers including multiple myeloma, chronic lymphocytic leukemia (CLL) and indolent B-cell non-Hodgkin lymphoma (NHL) for which the FDA has approved its use, and multiple myeloma. Importantly, BDM is highly effective in NHL patients who have failed conventional alkylator therapies [1], thus supporting the idea that BDM has different mechanisms of action. Despite the many years of clinical use of BDM, and demonstration of ability to alkylate DNA, its precise mechanism of action in cells remains obscure. A central theme regarding BDM is whether or not it causes a different cellular insult as compared to standard alkylating agents (reviewed in [2]). A previous report suggested that the type of DNA damage induced by BDM was different to that caused by alkylating agents [3]. For example, the treatment of the non-Hodgkin lymphoma cell line SU-DHL-1 with BDM led to a greater increase in the expression of several p53-responsive genes and DNA damage/repair genes when compared to other alkylators. The differences in the expression pattern of genes involved in DNA-damage stress response, apoptosis, cell cycle, mitosis and DNA replication between BDM and standard alkylating agents suggested that BDM elicits a different cytotoxic response that was mediated by a mechanism of action unlike that of other alkylating agents [3]. Mechanistic studies using human CLL and mantle cell lymphoma (MCL) cell lines [4] and CLL cells from patients [5] point to activation of classical apoptosis through mitochondrial perturbation, Bax induction and activation, release of cytochrome C and subsequent caspase-3 activation after BDM treatment. Furthermore, by interrogating CLL and mantle cells from patients, Roue *et al.* found no correlation between p53 status and BDM cytotoxicity [4].

Less well studied is the connection between BDM and cell cycle progression. Myeloma cells treated with BDM at 10–30 µg/ml (equivalent to 25–76 µM) arrested in G2 [6], whilst SU-DHL-1 cells treated with BDM at 50 µM resulted in a modest S phase delay [3]. Whether the different cell cycle responses were due to idiosyncrasies of the cell lines or due to different concentrations of drug remains unclear. A major unaddressed question raised by these studies is whether cell cycle arrest induced by BDM is related to cell death. Here we set out to characterize the effect of BDM on cell cycle progression, DNA damage induction and repair, and cell viability.

## Materials and Methods

### Materials

Bendamustine, propidium iodide, chlorambucil, melphalan and Chk2 inhibitor II were all purchased from Sigma. The UCN-01 was generously provided by Kyowa Hakko Kirin Co., Ltd. and the National Cancer Institute, NIH.

### Cell lines and culture conditions

All cell lines were originally obtained from the ATCC and banked at the Fox Chase Cancer Center (FCCC) Cell Culture facility. Mycoplasma testing was conducted at FCCC prior to studies. The cell lines HeLa, PANC1, BxPC3, MCF7, MDA-MB-453 were grown in DMEM supplemented with 10% FBS, 2 mM glutamine and 1% penicillin, streptomycin and kanamycin (PSK). OVCAR 5 and 10 cells were grown in RPMI supplemented with 10% FBS, 2 mM glutamine and 1% PSK. U2932 were grown in RPMI supplemented with 15% FBS, 2 mM glutamine and 1% PSK. Wildtype and TDG −/− MEFs, generated as previously described [7], were grown in DMEM supplemented with 15% FBS, 2 mM glutamine, 1% PSK and sodium pyruvate. All cells were maintained at 37°C, 5% CO_2_.

### Cell viability and apoptosis assays

HeLa cells were seeded into 96 well plates at a density of 1500 cells per well. Cells were treated with BDM (3.125–200 µM) for 24 h. After this time cells were additionally treated either with UCN-01 (100 nM), Chk2 inhibitor (100 nM) or Gö6976 (200 nM) for an additional 24 h. MTS reagent (CellTiter 96® AQ_ueous_ Proliferation Assay, Promega) was added to each well for 4 h before the absorbance values at 490 nm was read. Experiments were conducted in triplicate, 3 independent times. Data presented are the average absorbance values, relative to control ± SD. For apoptosis determination, cells seeded and treated as stated above were stained with Guava Nexin Reagent ® (Millipore). Analysis was conducted using the Easycyte module and Cytosoft Software® (Millipore). Experiments were conducted 3 independent times, collecting 2000 events each time. Data shown is the average ± SD.

### Clonogenic assay

HeLa cells were seeded into 6 well plates at a density of 1000 cells per well. Cells were treated for 24 h. Cells were then treated with and without UCN-01 (100 nM) for 3 h. All drugs were washed out and cells were left to grow for approximately 10 days. Cells were fixed using acetic acid∶methanol∶H_2_O, (10∶10∶80), dried and stained with 0.4% crystal violet in 20% ethanol. Qualitative images of representative results are presented. For quantification, colonies were solubilized (1% acetic acid, 30% ethanol) and absorbance values at 595 nm were read. Values are expressed as a percentage relative to untreated cells (set to 100% ± SD). Experiments were conducted in triplicate, three independent times. Student's two-tailed unpaired *t* test was conducted for statistics.

### Metaphase spreads

Mitotic cells were collected from untreated and BDM at 50 µM or 200 µM+UCN-01 (100 nM) treatments. Cells for metaphase spreads were prepared as outlined by [8]. For visualizing, cells were dropped onto glass slides and stained with DAPI. Mitotic spreads were viewed and imaged using fluorescence microscopy. Images shown are representative of those observed.

### Electron microscopy

HeLa cells were seeded into 6 cm dishes, synchronized with thymidine (2 mM) and treated with BDM 50 µM, 200 µM for 18 h. Cells treated with UCN-01 (100 µM) for an additional 9 h to obtain mitotic cells were fixed with glutaraldehyde solution (3%, pH 7.4). Samples were prepared for electron microscopy analysis as previously described [9].

### Immunofluorescence

Cells were grown on coverslips, treated with the indicated drugs, fixed and stained as previously described [10]. To detect DNA damage induction and repair, antibodies against 53BP1 (Bethyl) and γ-H2AX (Upstate) were used with Alexa Fluor-conjugated secondary antibodies (Invitrogen). All cells were counter-stained with DAPI (Molecular Probes). Images were captured using a 40× or 100× objective mounted on an inverted microscope (Eclipse TE2000S; Nikon) with a CCD camera (Photometrics Cascade 512F; Roper Scientific) powered by NIS Elements AR (version 3.10) software (Nikon). For quantitative analysis, individual nuclei were identified using DAPI staining and used to define regions of interests (ROI), which were then transposed onto other channels. The sum intensity per nuclei from a minimum of 50 nuclei was averaged ± SEM.

### EdU incorporation

HeLa cells were treated with nocodazole for 14 h after which time mitotic cells were collected by mechanical shake-off. Cells were washed twice with medium and re-plated. BDM and EdU (10 µM) (Click-iT™ EdU imaging kit, Invitrogen) were added 2 h later, when the cells were fully adherent and still in G1, and incubated for an additional 12 h. Cells were fixed and stained according to the manufacturer's protocol. Quantification of EdU incorporation was performed as described for antibody detection.

### Time-lapse video microscopy

HeLa cells stably expressing green fluorescent protein (GFP)∶histone H2B were seeded into 6 well plates and synchronized in S phase using thymidine (2 mM). Thymidine was washed out and 11 h later, cells were treated with the indicated drugs for an additional 18 h. Where appropriate, UCN-01 (100 nM) was added to wells before being supplemented with HEPES (25 mM), layered with mineral oil (Sigma) and placed into a housing chamber which maintained the temperature at 37°C. Using a Nikon TE2000 microscope (Nikon) controlled by MetaMorph software (Molecular Devices), bright field and fluorescent images were captured every 5 minutes for up to 48 h. Individual movies were analyzed manually and a minimum of 100 cells per treatment scored for the indicated measurements. For montages, selected frames representing different cell morphologies are presented.

### DNA content analysis via flow cytometry

Following indicated drug treatments, cells were fixed with 70% ethanol. For analysis, cells were stained with propidium iodide, RNase A and sodium citrate. Cells were incubated at 37°C for 30 minutes before being analyzed using a Becton Dickinson single laser three-fluorescence FACScan flow analyzer and Cell Quest software (Becton Dickinson). DNA content was acquired from 10,000 events and FlowJo software was used to process the data.

### Statistical analysis

For statistical analyses, Student's two-tailed *t* test was conducted.

## Results

### BDM displays concentration-dependent effects on cell cycle response

**Figure 1:**
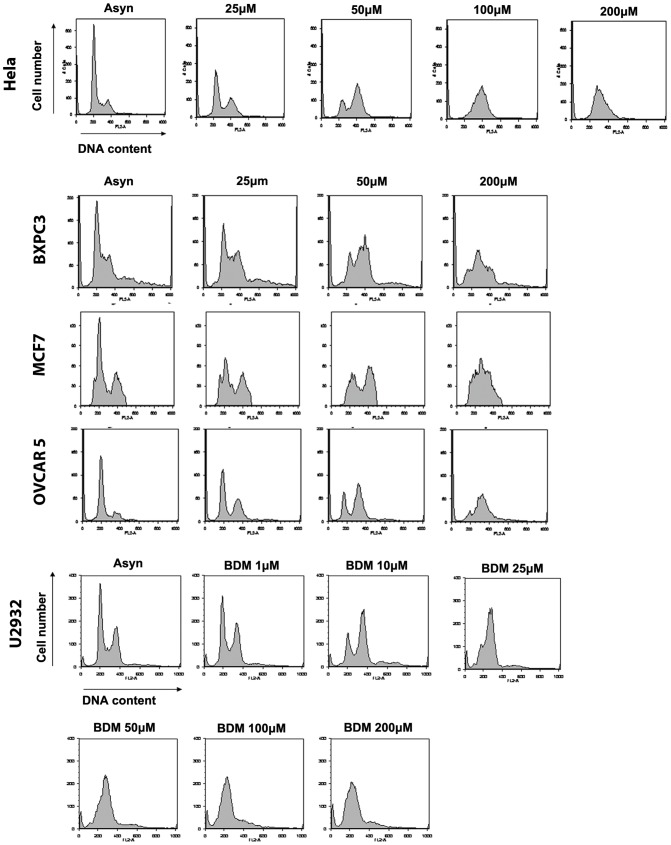
Cell cycle perturbations induced by bendamustine are a widespread phenomenon in cancer cell lines. HeLa, BXPC3, MCF7, OVCAR 5 and U2932 cells were treated with bendamustine at the indicated concentrations for 24 h. Cell cycle profiles were determined using FACS analysis.

HeLa cells synchronized in G1 by release from a mitotic shakeoff were treated with increasing concentrations of BDM and their cell cycle profiles were assessed 24 h later. Figure 1 shows that HeLa cells treated with increasing doses of BDM (25–100 µM) accumulated at a 4 N DNA content indicating a G2/M delay. In contrast, cells treated with 200 µM BDM were arrested in S phase. We next confirmed the dose dependent effects of BDM on the cell cycle in a wide panel of cancer cell lines. Cell lines derived from pancreatic (PANC1 and BXPC3 cells), breast (MCF-7 and MDA-MB-435), and ovarian (OVCAR 5 and 10) cancers responded similarly to HeLa cells (Fig. 1 and Figure S1) in that 50 µM BDM caused a G2 arrest while 200 µM BDM resulted in an S phase arrest. The concentrations of BDM used here are consistent with those previously used against a number of epithelial derived cell lines [11], [12], but are generally higher than concentrations used for leukemic cell lines [12]. To test if this was a feature of adherent cell lines, we treated the U2932 cells (B cell lymphoma) with BDM. As seen with the adherent cell lines, U2932 cells also exhibited a dose dependent cell cycle response, but at a lower concentration: 10 µM induced a G2 arrest, 50 µM induced an S phase arrest. The combined data demonstrate that BDM induces a dose-dependent effect on cell cycle progression in multiple cell lines.

We measured DNA synthesis to further assess the effects of BDM on cell cycle progression. HeLa cells synchronized by mitotic shake-off were treated in G1 with either 50 or 200 µM BDM in the presence of EdU (10 µM) for 12 h. Cells treated with 50 µM BDM incorporated essentially the same amount of EdU (98.0±4.1%) as untreated cells (100±4.8%), while cells treated with 200 µM BDM incorporated only 64.7±2.6% as compared to untreated cells (p = 1.1×10^−10^). This suggests that 200 µM BDM reduces the efficiency of replication, consistent with the delay in S phase progression. Although replication was not grossly affected by 50 µM BDM, the G2 delay may reflect accumulation of replication errors that did escape the S phase checkpoint.

### Bendamustine induces both repairable and irreparable DNA damage

**Figure 2:**
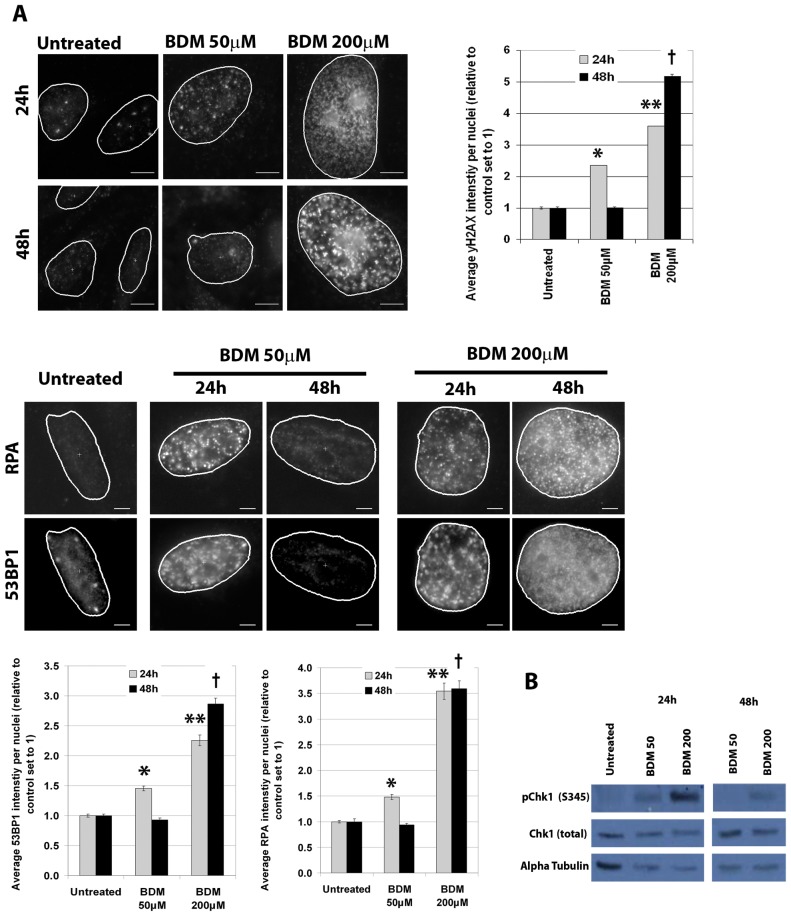
Bendamustine induces both repairable and irreparable DNA damage. HeLa cells were treated for 24 or 48 h continuous treatment with either 50 or 200 µM BDM or 24 h followed by 24 h in the absence of drugs. **A.** Immunofluorescence analysis was performed to identify γ-H2AX, 53BP1 or RPA foci. Quantification of the average fluorescence per nucleus (nucleus outlined) is shown on the right. For γ-H2AX: *P = 8.9×10^−28^ vs. untreated 24 h; ** P = 2.1×10^−31^ vs. untreated 24 h; †P = 5.2×10^−45^ vs. untreated 48 h. For 53BP1: *P = 4.3×10^−20^ vs. untreated 24 h; ** P = 4.2×10^−41^ vs. untreated 24 h; †P = 2.0×10^−57^ vs. untreated 48 h. For RPA: *P = 2.5×10^−16^ vs. untreated 24 h; ** P = 2.8×10^−52^ vs. untreated 24 h; †P = 3.3×10^−58^ vs. untreated 48 h. **B.** Lysates were probed to determine p-Chk1 (Ser345). Total Chk1 and alpha tubulin were used to determine loading.

The cell cycle arrest observed in Figure 1 was likely a result of BDM-induced DNA damage, activating the DNA damage checkpoint pathway. We therefore examined phospho-H2AX (Ser 139), 53BP1 and replication protein A (RPA), three different DNA damage response proteins, as a direct measure of BDM-induced DNA damage. We found that BDM induced H2AX foci, reflecting accumulation at sites of DNA damage (Figure 2A). We observed a concentration-dependent increase in γ-H2AX foci after 24 h treatment. However, after 48 h of treatment with 50 µM BDM γ-H2AX foci was reduced to levels seen in vehicle-treated cells (Figure 2A). The reduction in H2AX was likely a result of efficient DNA repair. However, after 48 h treatment with 200 µM BDM the level of H2AX foci was not reduced. We next confirmed these results by staining for 53BP1 and RPA. After 24 h treatment we observed a dose-dependent increase in foci formation, as seen with H2AX foci. Furthermore, after 48 h with 50 µM BDM levels of 53BP1 and RPA were reduced to control levels. In contrast, foci formation persisted after 48 h continuous treatment with 200 µM BDM.

We next compared the cell cycle profiles of cells treated with 50 µM or 200 µM for 24 h, 48 h continuously or for 24 h with BDM, followed by 24 h in the absence of drug. Figure S2 shows the expected cell cycle arrest after treatment with 50 µM BDM for 24 h, but after another 24 h in the presence or absence of BDM, cells displayed a normal cell cycle profile. This is consistent with the efficient repair (lack of foci) of DNA damage at 50 µM BDM. In contrast, the cell cycle arrest induced by 200 µM BDM was maintained following 48 h continuous treatment, and even 24 h after BDM was washed out, this did not reverse the arrest. This finding is consistent with the persistent DNA damage foci at 200 µM BDM.

Since Chk1 is a key regulator of cell cycle response following cellular exposure to replication stress, we assessed Chk1 phosphorylation (Ser-345) as a biochemical readout for cellular response to BDM. We observed that Chk1 was dose-dependently phosphorylated by BDM after 24 h treatment (Fig. 2B). Importantly, after 48 h no signal was observed in cells treated with 50 µM BDM as would be predicted from the IF and cell cycle data. However, detectable pChk1 was still present in cells treated with 200 µM BDM, confirming these cells were still checkpoint arrested. Taken together, these data suggest that DNA damage induced by 50 µM BDM is efficiently repaired, but at 200 µM BDM the effects on cell cycle and DNA damage were irreversible.

### Involvement of base excision repair in bendamustine-induced DNA damage

**Figure 3:**
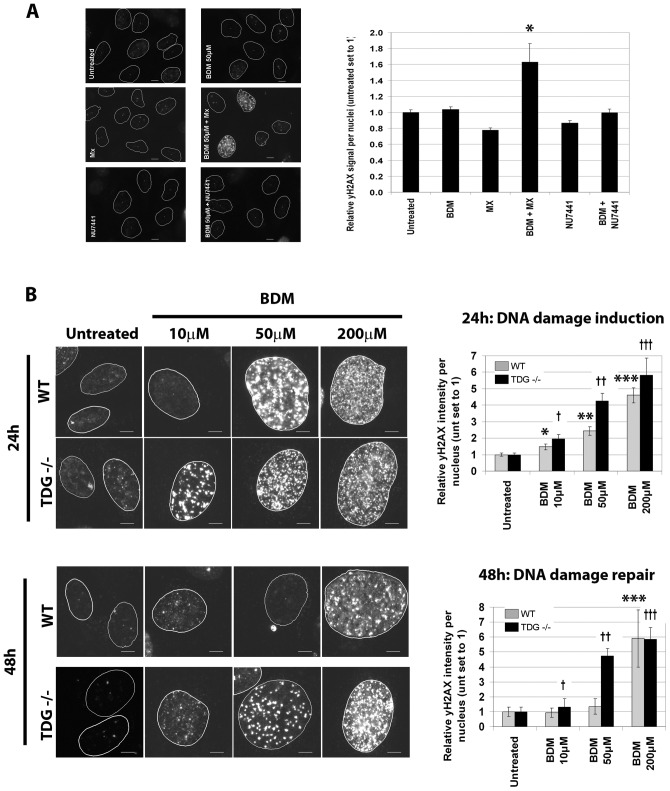
Involvement of base excision repair in bendamustine-induced DNA damage. **A.** Immunofluorescence analyses were performed to detect remaining γ-H2AX foci after 48 h continuous treatment with 50 µM BDM in the presence or absence of either methoxyamine (MX) (6 mM) or the DNA PK inhibitor NU7441 (10 µM). Representative images are shown (left) along with quantitative analysis (right). Average values ± SD are shown. *P<0.005 vs. BDM alone. **B.** DNA damage induction and repair was conducted in assessed MEFs (*Tdg* ^+/+^ and *Tdg* ^−/−^) after treatment with BDM at 50 or 200 µM BDM for 24 h or 48 h. Representative images are shown, with nuclei outlined (circles) based on DAPI staining. Average γ-H2AX signal per nucleus ± SD is quantified (right). 24 h: *p = 0.009, **p = 2.0×10^−8^, ***p = 4.7×10^−18^ vs. untreated WT; †p = 0.00015, **p = 2.8×10^−11^, ***p = 5.2×10^−10^ vs. untreated TDG −/−. 48 h: ***p = 4.1×10^−10^ vs. untreated WT; †p = 0.009, **p = 1.4×10^−16^, ***p = 1.9×10^−16^ vs. untreated TDG −/−.

A previous study showed that BDM-induced cell death was enhanced by an inhibitor of base excision repair (BER) [3]. We therefore sought to test the involvement of BER in whether BDM-induced damage. We targeted the essential BER protein apurinic/apyrimidinic endonuclease (APE1) by using the pharmacological inhibitor methoxyamine (MX) [13]. We treated HeLa cells with BDM 50 µM in the presence and absence of methoxyamine and assessed DNA damage. Untreated or cells treated with BDM 50 µM for 48 h had little γ-H2AX foci, as expected (from figure 2A). However, addition of MX to BDM-treated cells resulted in the retention of significantly higher levels of γ-H2AX foci than BDM alone or control cells (Fig. 3A). In contrast, using NU7441, a DNA-PK inhibitor that does not block BER, did not affect repair of BDM-induced damage.

Thymine DNA Glycosylase (TDG) is a prominent BER enzyme whose levels are regulated during the cell cycle [14]. For this reason, we tested its involvement in BDM-induced damage. We studied an isogenic pair of mouse embryonic fibroblasts (MEFs) that were either wild type (*Tdg* ^+/+^) or with a genetic ablation of the *Tdg* gene (*Tdg* ^−/−^) [7]. Not only did we observe that *Tdg* ^−/−^ MEFs were more sensitive to 10 µM BDM than wild type MEFs, showing increased γ-H2AX staining after 24 h of treatment, but they also had a reduced ability to repair the DNA damage after 48 h continuous treatment, as compared to wild type MEFs (Fig. 3B).

### Chk1 inhibition accelerates bendamustine-induced cell death

**Figure 4:**
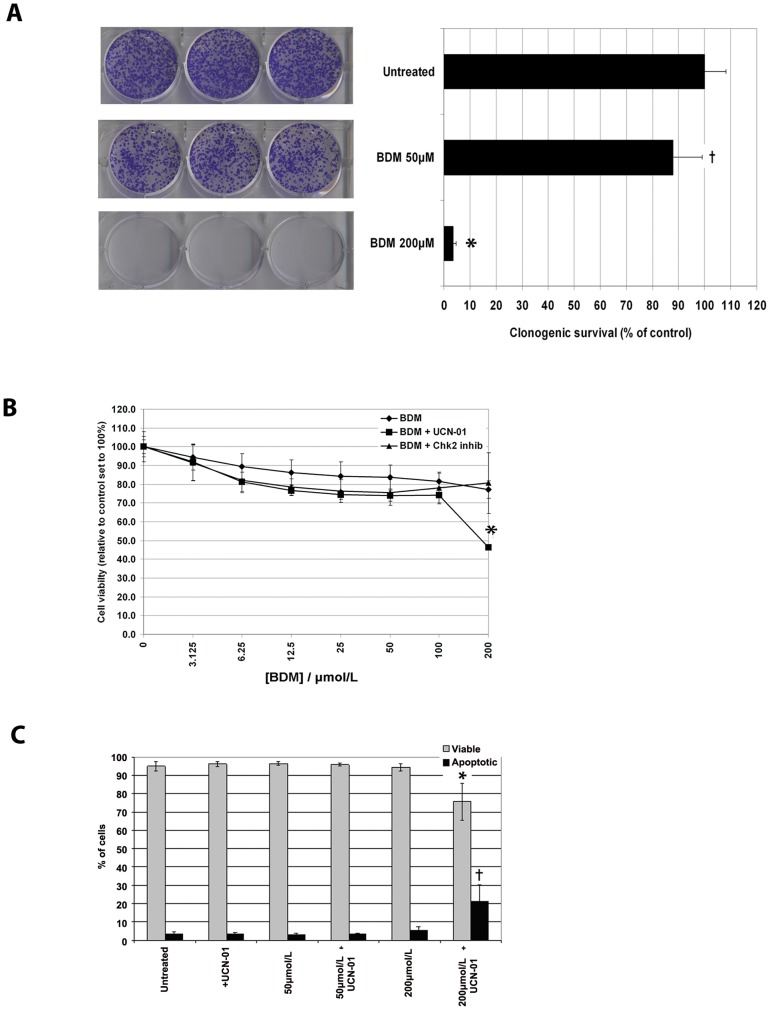
Chk1 inhibition accelerates bendamustine-induced cell death. **A.** Clonogenic assays were performed to assess cell survival following BDM and Chk1 inhibition. HeLa cells were treated with BDM (50 or 200 µM) for 24 h. Cells were grown for ∼10 days before being fixed and stained. The data presented are the mean absorbance value (O. D. 595 nm) relative to untreated cells, which is set to 100%. Each bar graph represents the average of 3 individual experiments performed in triplicate ± SD. †P<0.05 or *P<0.0001 or vs. untreated cells. **B.** Cell viability assessed by MTS assay was performed. HeLa cells were treated with BDM (3.125–200 µM) for 24 h. After this time, appropriate wells were co-treated with UCN-01 (100 nM) or Chk2 inhibitor (100 nM) for an additional 24 h. Data presented is the mean of 3 individual experiments performed in triplicate. Cell viability is expressed as a percentage of untreated cells ± SD. *P<0.0001 vs. 200 µM BDM alone. **C.** The percentage of apoptotic cells following indicated drug treatments was determined using Guava Nexin Reagent™. Data presented is the average of 3 individual experiments ± SD. *P<0.01 vs. untreated viable cells; †P<0.01 vs. untreated apoptotic cells.

Since recent pharmacokinetic studies demonstrated that BDM is rapidly metabolized in patients [15], [16], we modified the clonogenic assay to assess how transient exposure to BDM affects long term survival. HeLa cells were treated for 24 h with BDM at 50 µM and 200 µM after which time drugs were washed out, replaced with fresh medium and allowed to recover. As shown in Figure 4A, colony formation between control cells versus cells treated with 50 µM BDM was marginally reduced (control 100±8.1% versus 87.8±11.2%; p<0.05). However, cells treated with BDM 200 µM showed a dramatic reduction in the number of colonies formed (control 100±8.1% versus 3.5±1.3%; p<0.0001). Thus, concentrations of BDM (200 µM) that induced irreparable DNA damage and a sustained cell cycle arrest leads to efficient cell death. However, given the timescale reflected in the clonogenic assay after treatment with BDM 200 µM (∼10 days), it is possible cells may acquire resistance to the drug and escape death. Since BDM 200 µM induced a sustained cell cycle arrest, we hypothesized that abrogating the checkpoint would accelerate killing by BDM. Figure 4B shows that BDM at any concentration (3.125–200 µM) is not particularly effective at killing HeLa cells after 48 h of continuous treatment. We next successively treated cells with BDM (3.125–200 µM) for 24 hours and then added UCN-01 (100 nM) for another 24 h, before assaying for cell viability. UCN-01 did not sensitize cells pre-treated with BDM up to 100 µM to killing. In contrast, viability was significantly reduced in cells pre-treated with 200 µM BDM followed by UCN-01. Similar sensitization was observed with Gö6976, another compound that inhibits Chk1 (data not shown). A pharmacological inhibitor of Chk2 did not sensitize to killing by any concentration of BDM in the same range.

Having demonstrated HeLa cell death was increased after BDM 200 µM+UCN-01, but not BDM 50 µM+UCN-01, we next examined the nature of killing. Cells were stained to discriminate between necrotic or apoptotic cell death. Notably, only cells treated with BDM 200 µM+UCN-01 or Gö6976 exhibited an increase in the percentage of apoptotic cells (p<0.01 vs. control) (Figure 4C and data not shown). It should be noted that UCN-01 alone had no discernable effects on cell viability in any of the assays performed. Taken together, these findings demonstrate that forcing cells to overcome the checkpoint induced by 200 µM BDM, but not 50 µM, BDM causes cells to die more effectively compared to cells treated with BDM alone.

### Overcoming bendamustine-induced checkpoint arrest via Chk1 inhibition forces cells into premature mitosis

**Figure 5:**
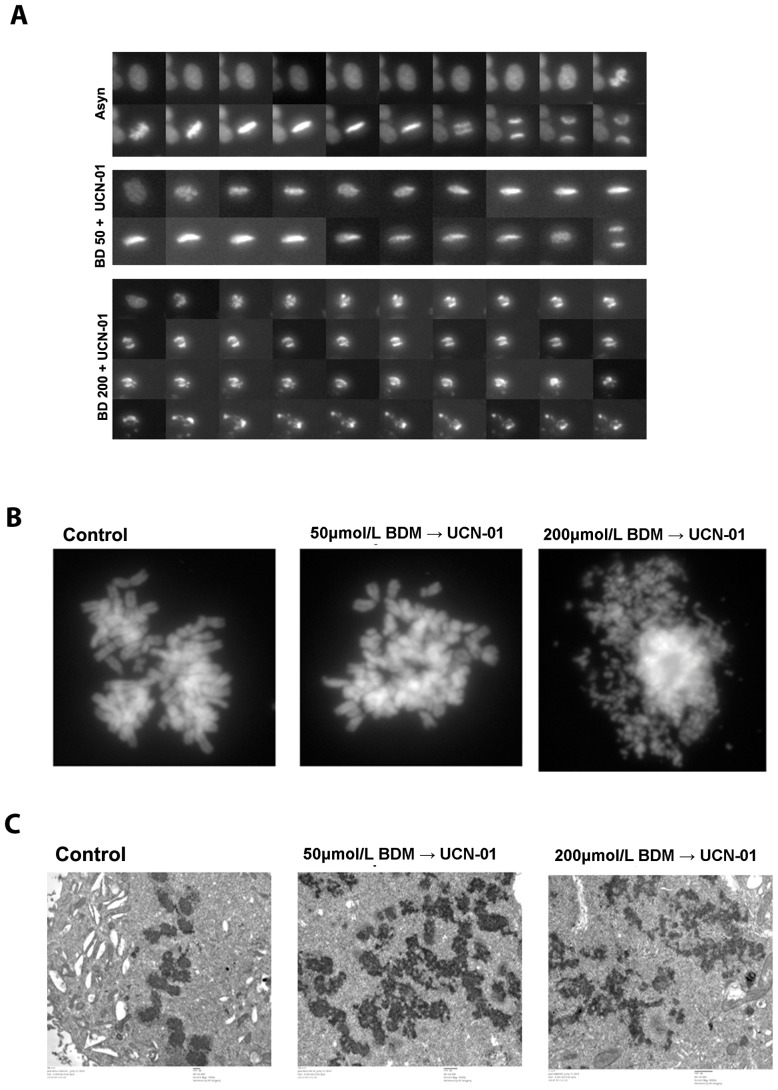
Overcoming bendamustine-induced checkpoint arrest via Chk1 inhibition forces cells into premature mitosis. HeLa cells stably expressing GFP∶histone H2B were used for live cell video-microscopy. **A.** Representative montage of cells progressing through mitosis after mock treatment (upper panel), BDM at 50 µM (middle) or 200 µM (lower) followed by UCN-01 addition. **B.** Mitotic cells were fixed for metaphase spreads and dispersed onto glass slides, allowed to dry and then stained with DAPI. Metaphases were visualized using fluorescence microscopy. Images shown are representative of metaphases observed under each experimental condition. **C.** Representative electron micrographs of mitotic cells generated from untreated, 50 µM or 200 µM BDM+UCN-01 treatments.

To gain insight as to how inhibition of Chk1 accelerated cell killing by 200 µM BDM, the fates of individual cells were tracked by time-lapse microscopy. HeLa cells stably expressing GFP∶Histone H2B were treated with vehicle, 50 µM or 200 µM of BDM for 24 h before UCN-01 (100 nM) was added. The time-lapse data showed that vehicle treated cells entered and progressed normally through mitosis (average time of mitosis = 52.5±16.7 minutes) (Figure 5A, upper panel and Fig. S2). 50 µM BDM caused a reduction in the percentage of cells entering mitosis over a 24 h period (Fig S2), consistent with the G2 arrest observed by FACS analysis. Addition of UCN-01 abrogated the arrest and cells began to enter mitosis 4 hours later (Figure 5A, middle panel). While chromosome congression and alignment appeared normal, cells were nevertheless delayed at metaphase for an average of 89±64.7 minutes, as compared to controls that exited 37.5±16.7 minutes after metaphase alignment. Upon exiting mitosis, a fraction of cells exhibited chromosomal aberrations, such as lagging chromosomes (Fig S3). Similarly, cells treated with 200 µM BDM had a reduced mitotic index, consistent with the cell cycle data. Inhibition of Chk1 induced mitotic entry but, unlike at 50 µM BDM, the mitotic figures of these cells were highly abnormal (Figure 5A, bottom panel). For example the chromosomes failed to align at the spindle equator, and cells rarely exited mitosis but instead died without exiting. The few cells that did exit formed multinucleated cells. Thus, cells forced into mitosis after 50 µM BDM+Chk1 inhibition progress through mitosis relatively normally. In contrast, cells forced into mitosis after 200 µM BDM+Chk1 inhibition generate extremely abnormal mitotic figures, with the majority failing to exit and instead dying in mitosis (Fig S3).

We next examined the chromosome morphology of cells that were forced to enter mitosis after treatment with 50 µM or 200 µM BDM+UCN-01 in greater detail. Metaphase spreads obtained from control and BDM 50 µM+UCN-01 treated cells showed no obvious gross structural defects (Figure 5B). In contrast, the metaphase spreads of BDM 200 µM+UCN-01 samples were extremely abnormal. Instead of forming discrete chromosomes, they appeared ‘pulverized’, a phenotype indicative of extensive chromosome fragmentation [17].

This is consistent with early studies that showed that S phase cells when fused with mitotic cells underwent premature chromosome condensation (PCC) that resulted in a pulverized morphology [18]. We therefore used electron microscopy to examine the ultra-structure of chromosomes after different drug treatments (Fig. 5C). In control cells, as well as in mitotic cells obtained after treatment with BDM 50 µM+UCN-01, intact chromosomes were clearly visualized. However, in mitotic cells generated by the treatment with BDM 200 µM+UCN-01 chromosomes were highly fragmented.

## Discussion

The studies described here show that BDM can induce an S or G2 cell cycle arrest depending on its concentration. This effect was not idiosyncratic of HeLa cells, as it was observed in cell lines derived from pancreatic, breast and ovarian cancers, at similar concentrations of BDM. We found that only a ∼four-fold difference (50 and 200 µM for adherent cells, 10 and 50 µM for lymphoblast) in concentration can change the cell cycle response from a G2 to an S phase arrest. The maximum concentration of BDM used in this study, 200 µM, to elicit cell cycle perturbations in ‘solid’ cancer cell lines are consistent with the IC50 values obtained in previous studies [12]. While 200 µM is ∼8–13 times higher than clinically achieved [16], [19], the lymphoma cell line U2932 did show the same dose-dependent cell cycle arrest at more clinically relevant concentrations of BDM; cells treated with 10 µM BDM resulted in a G2 arrest, while 25 µM BDM resulted in an S phase arrest. These findings suggest that the hematologic malignancies may be more sensitive than cell lines derived from solid tumors to BDM, as previously observed [12]., and that the therapeutic value of BDM in the clinic may be associated with the induction of S phase arrest.

The studies presented here do not resolve the whether BDM acts as an alkylator, as an anti-metabolite or a combination of the two. However, we show that BDM at 50 µM and 200 µM has distinct effects on cell cycle and induction of DNA damage. Notably, the repair capacity after treatment with 50 µM and 200 µM is distinct: after of 48 h continuous exposure, cells treated with 50 µM BDM exhibited no discernable markers of DNA damage, indicating that the damage was efficiently repaired. Moreover, cells were no longer arrested in G2 but were cycling normally, further suggesting repair of DNA damage. In contrast, cells treated with 200 µM BDM for 48 h failed to repair the damage, as shown by persistent γH2AX foci and sustained cell cycle arrest. Thus, these data support the notion that DNA damage induced by 200 µM BDM is repaired much less efficiently than at 50 µM. Furthermore, these findings are consistent with observations that not only does BDM induce more DNA double strand breaks as compared to the alkylating agents cyclophosphamide and BCNU, but also the BDM-induced damage is not repaired as efficiently as these two drugs [11]. Our studies are unable to distinguish between several distinct, but not necessarily mutually exclusive models that may account for the generation of irreparable DNA damage. The most simplistic explanation is that the amount of damage induced at 200 µM may quantitatively overwhelm the repair capacity of the cell to an extent that it slows down DNA replication. Another possibility is that 200 µM BDM may induce damage that differs qualitatively from that achieved with 50 µM BDM. A third possibility suggests that 200 µM BDM may inhibit proteins that are essential for repair. It is possible that the purine-like ring structure within BDM inhibits key proteins that require nucleotides for their activity (DNA polymerases, kinases, helicases). For example, caffeine contains a purine ring and can inhibit the activity of ATM and ATR, key kinases involved in double strand break repair [20]. Finally, BDM may be metabolized differently at the two doses such that only the higher concentration of BDM (200 µM) is present long enough to cause irreversible damage to DNA. Further studies are needed to test these hypotheses.

Our study highlights the potential use of combining BDM with inhibitors of DNA repair for therapeutic relevance. Using a combination of cell lines genetically ablated for TDG, as well as pharmacological inhibition of BER, indicates that at least a fraction of BDM-induced damage is repaired by BER. However, we note that repair is attenuated and not completely ablated, suggesting the involvement of other pathways. It is possible that mismatch repair that is known to mediate cytotoxicity of alkylating agents [21] may be involved in the repair of BDM-induced damage. Nonetheless, previous findings demonstrating that BDM-induced cytotoxicity was enhanced by co-treating cells with methoxyamine [3] suggest that combinatorial treatment of BDM with inhibitors of DNA repair may translate to therapeutic efficacy.

The difference in DNA damage repair efficiency between the two concentrations of BDM may help explain our findings on short term and long term cell viability. Our findings imply that short term exposure (24–48 h) to BDM at 50 µM induces transient DNA damage which the cell eventually repairs, leading to no discernable long-term effects as determined by clonogenic survival assays. At 200 uM BDM, damage is not repaired and a 95% reduction in clonogenic survival is observed.

We are currently unsure as to how cells die after treatment with 200 µM BDM but the short term assays provide clues. Using short-term assays, 48 h continuous exposure to BDM up to 200 µM did not reduce cell viability as compared to control treatment. Thus, the time and concentration of BDM is sufficient to activate cell cycle arrest but not to trigger apoptosis. Cell cycle arrest with 200 µM BDM presented us the opportunity to test the effect of Chk1 inhibition. Indeed, combining Chk1 inhibitors with conventional chemotherapy has proved beneficial in pre-clinical models of pancreatic cancer [22]. We showed that the inhibition of Chk1, but not Chk2, enhanced killing of BDM-treated HeLa in a short term assay. The ineffectiveness of Chk2 inhibitors was previously reported in a multiple myeloma cell line, despite the fact that BDM treatment activated Chk2 [6]. It is pertinent to note that sensitization by Chk1 inhibitors only occurred with 200 µM of BDM, the dose that elicited an S phase arrest. Using a combination of live-cell video-microscopy, metaphase spreads and EM, we show that the majority of the cells exhibiting extremely abnormal mitoses end up dying in mitosis. By killing cells in mitosis, this combinatorial treatment could reduce the number of cells that divide, survive, and potentially become refractory to treatment. Thus, treatment of cancers with BDM in combination with Chk1 inhibitors may provide a novel therapeutic strategy that warrants further investigation; however, this would still critically depend on the concentration of BDM that eliciting an S phase arrest, rather than a G2 arrest in order to kill cancer cells.

## Supporting Information

Figure S1 **BDM-induces cell cycle arrest in multiple cell lines.** Indicated cell lines were treated with increasing concentration of BDM for 24 h. Cell cycle profiles were determined using FACs analysis.
(TIF)

Figure S2 **Bendamustine concentration-dependent reversible and irreversible cell cycle arrest.** Hela cells treated with 50 or 200 µM BDM for 24, 48 or 24 h in the presence of drug followed by 24 h in the absence of drug (24 h→24 h wash out (w/o) were analyzed for cell cycle distribution using FACS analysis.
(TIF)

Figure S3 **Forced entry mitosis results in aberrant mitosis.** **A.** Quantification from the movie analysis was performed to measure the of the average time taken (minutes) to enter and exit mitosis ± SD (left panel) and the percentage of mitotic cells that displayed defects including lagging chromosomes or improper chromosome condensation (right panel). **B.** Quantification of the percentage of cells that enter mitosis of die in interphase after drug treatments (left panel), and measuring the fate of cells that enter mitosis (right panel).
(TIF)
