# Supplementary figures and images for: Dose Dependent Effects on Cell Cycle Checkpoints and DNA Repair by Bendamustine
Source: PLoS One. 2012 Jun 29;7(6):e40342. doi: 10.1371/journal.pone.0040342 (PMC3386996; doi:10.1371/journal.pone.0040342)

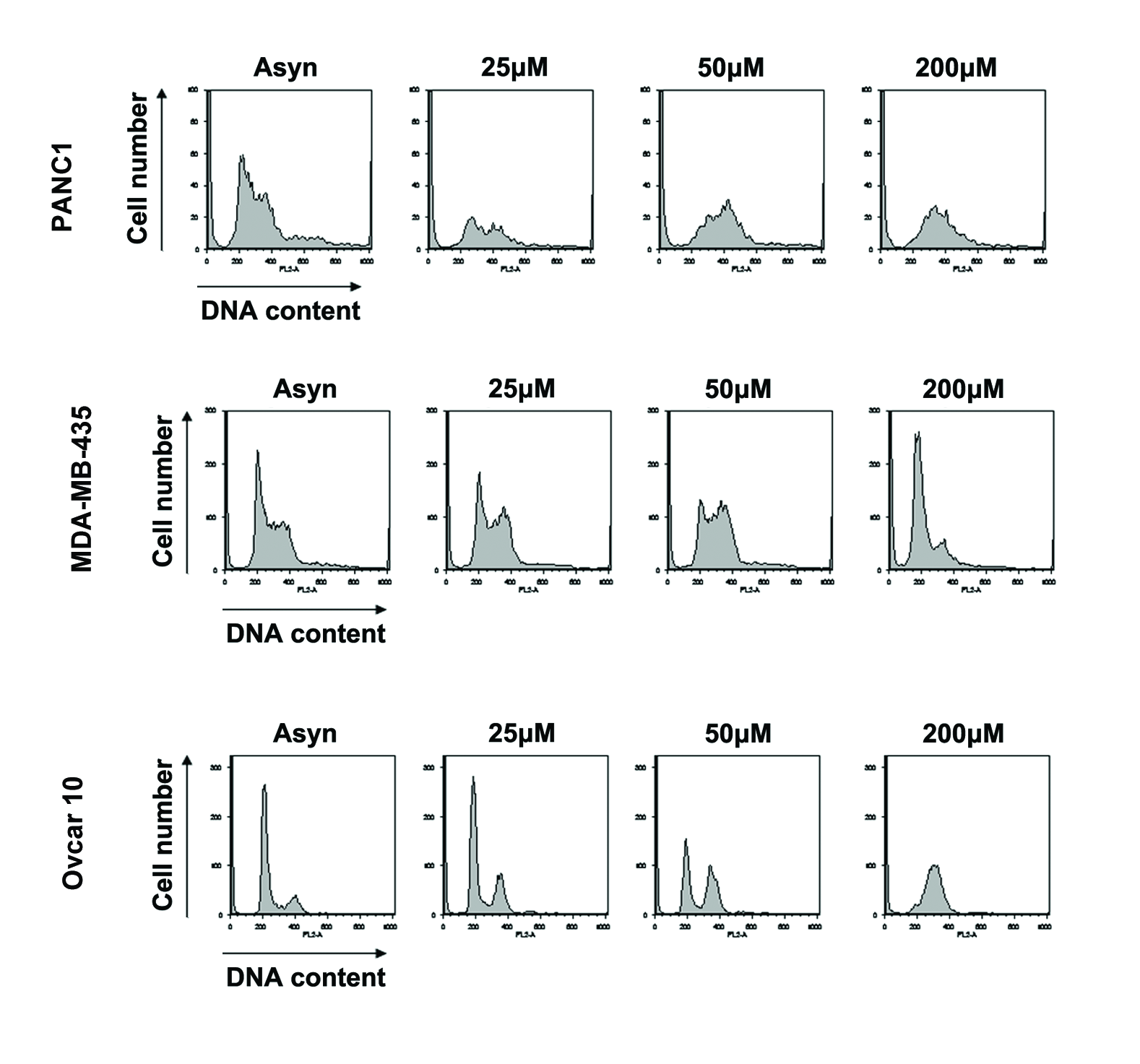

Supplement: Figure S1 — BDM-induces cell cycle arrest in multiple cell lines. Indicated cell lines were treated with increasing concentration of BDM for 24 h. Cell cycle profiles were determined using FACs analysis. (TIF) [file pone.0040342.s001.tif]

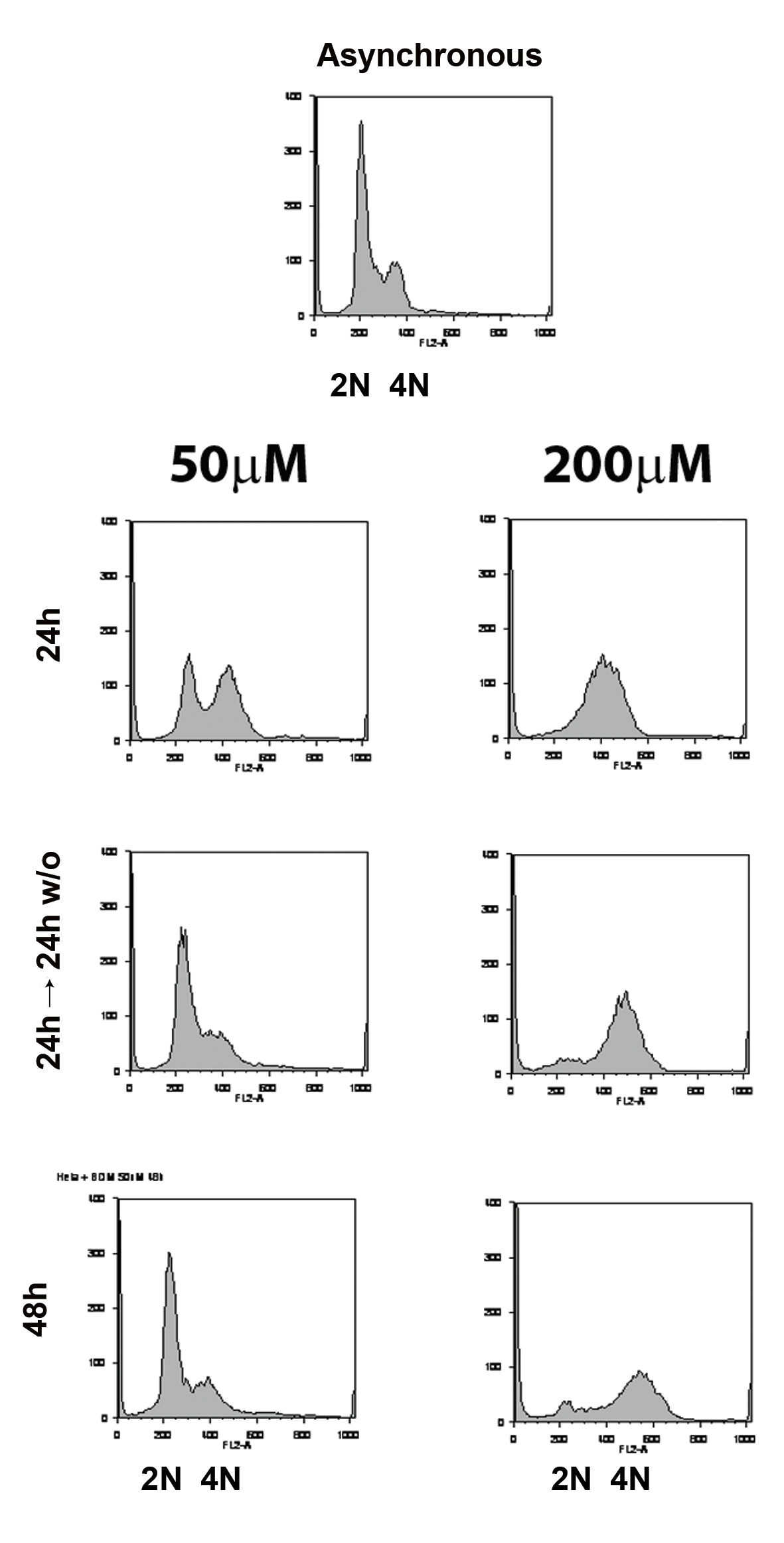

Supplement: Figure S2 — Bendamustine concentration-dependent reversible and irreversible cell cycle arrest. Hela cells treated with 50 or 200 µM BDM for 24, 48 or 24 h in the presence of drug followed by 24 h in the absence of drug (24 h→24 h wash out (w/o) were analyzed for cell cycle distribution using FACS analysis. (TIF) [file pone.0040342.s002.tif]

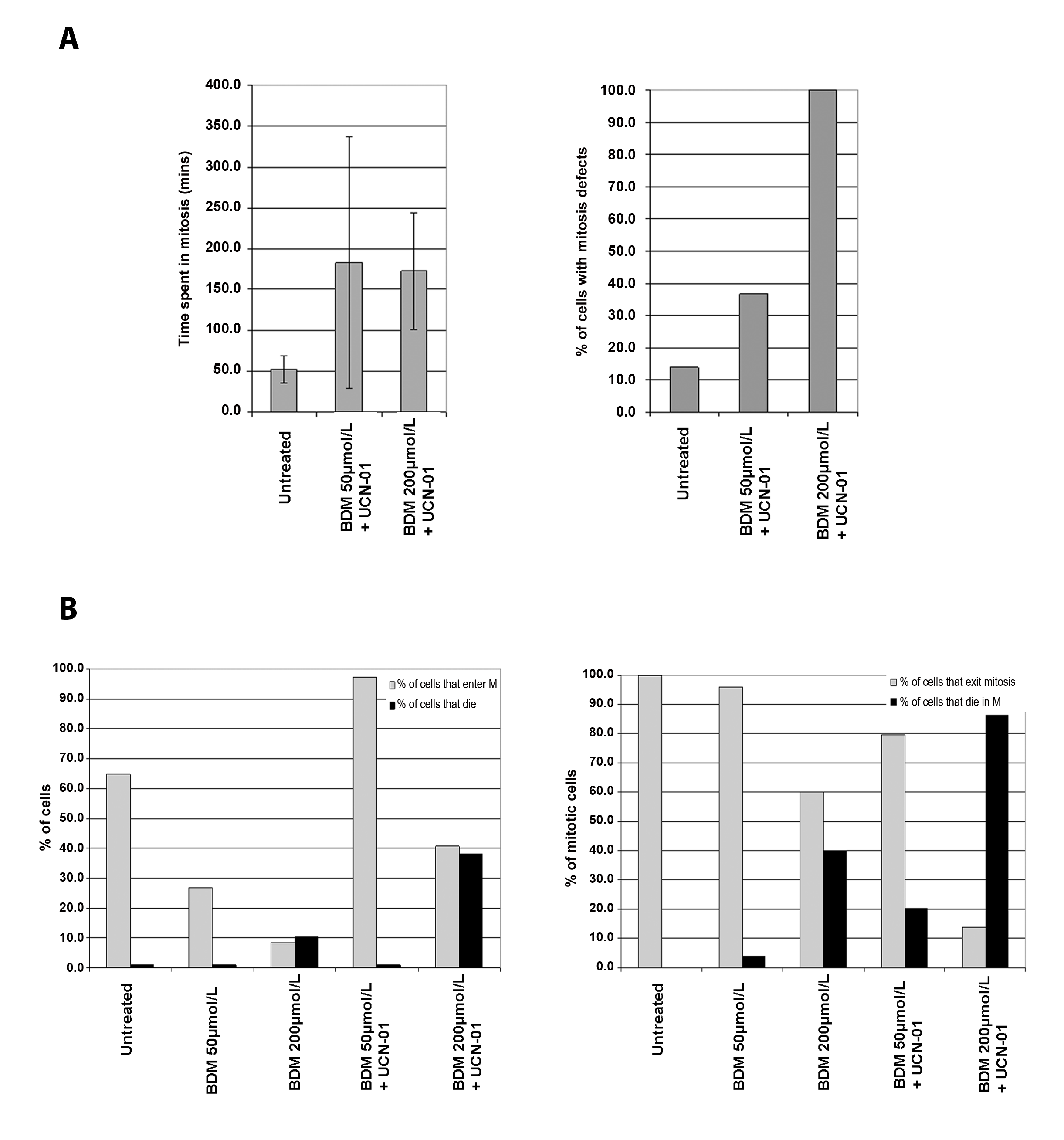

Supplement: Figure S3 — Forced entry mitosis results in aberrant mitosis. A. Quantification from the movie analysis was performed to measure the of the average time taken (minutes) to enter and exit mitosis ± SD (left panel) and the percentage of mitotic cells that displayed defects including lagging chromosomes or improper chromosome condensation (right panel). B. Quantification of the percentage of cells that enter mitosis of die in interphase after drug treatments (left panel), and measuring the fate of cells that enter mitosis (right panel). (TIF) [file pone.0040342.s003.tif]
